# Evolving Surgical Approaches to Bicuspid Aortic Valve Associated Aortopathy

**DOI:** 10.3389/fcvm.2019.00019

**Published:** 2019-03-04

**Authors:** Ali Fatehi Hassanabad, Christopher M. Feindel, Subodh Verma, Paul W. M. Fedak

**Affiliations:** ^1^Section of Cardiac Surgery, Department of Cardiac Sciences, Cumming School of Medicine, Libin Cardiovascular Institute of Alberta, University of Calgary, Calgary, AB, Canada; ^2^Division of Cardiac Surgery, Peter Munk Cardiac Centre, Toronto General Hospital, University Health Network, Toronto, ON, Canada; ^3^Division of Cardiac Surgery, St Michael's Hospital, Toronto, ON, Canada; ^4^Martha and Richard Melman Family Bicuspid Aortic Valve Program, Division of Cardiothoracic Surgery, Bluhm Cardiovascular Institute, Northwestern University, Chicago, IL, United States

**Keywords:** bicuspid aortic valve, bicuspid aortopathy, clinical guidelines, surgical approaches, clinical and surgical management

## Abstract

Bicuspid aortic valve (BAV) is the most common congenital cardiac pathology which results from the fusion of two adjacent aortic valve cusps. It is associated with dilatation of the aorta, known as bicuspid valve-associated aortopathy or bicuspid aortopathy. Bicuspid aortopathy is progressive and is linked with adverse clinical events. Hence, frequent monitoring and early intervention with prophylactic surgical resection of the proximal aorta is often recommended. Over the past two decades resection strategies and surgical interventions have mainly been directed by surgeon and institution preferences. These practices have ranged from conservative to aggressive approaches based on aortic size and growth criteria. This strategy, however, may not best reflect the risks of important aortic events. A new set of guidelines was proposed for the treatment of bicuspid aortopathy. Herein, we will highlight the most recent findings pertinent to bicuspid aortopathy and its management in the context of a case presentation.

*CASE: A 53 year-old, active and otherwise healthy male teacher has a known congenital bicuspid aortic valve, with fusion of the left and right aortic cusps. On recent transthoracic echocardiography, moderate aortic regurgitation and mild dilation of the left ventricle is present: Left Ventricle End Systolic Diameter (LVESD) of 50 mm. Systolic function is preserved. Moreover, a CT scan of the aorta demonstrates a mildly dilated aortic root (4.3 cm), a largely aneurysmal mid-ascending aorta (5.2 cm), and a normal aortic arch. Of note, the ascending aorta has increased in size by 5 mm over the past year. In the same time period, the patient has noticed a slight decrease in exercise capacity, but is otherwise asymptomatic with a low surgical risk status*.^1^

In your office, the patient inquires whether surgery is now indicated for his aorta?

## What is Bicuspid Aortic Valve Aortopathy?

**Figure 1:**
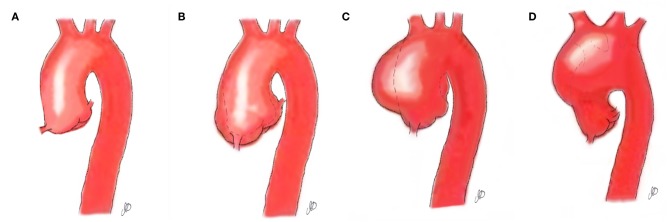
Aortopathy patterns seen in patients with bicuspid aortic valve. **(A)** Normal aorta; **(B)** Aortopathy affecting the aortic root; **(C)** Aortopathy affecting the ascending aorta; **(D)** Aortopathy affecting the aortic arch.

Bicuspid aortic pathology, also referred to as “bicuspid aortopathy,” is a common finding in patients with bicuspid aortic valve (BAV), with thoracic aortic dilation noted in ~40% of patients in referral centers (1) (Figure 1). The specific pattern of aortopathy can be variable between patients, resulting in heterogeneous clinical phenotypes. Aortopathy can be present in all aortic segments or more often, isolated to the aortic root (sinus segments), tubular ascending aorta, or proximal aortic arch. As in the case example, the majority of patients will present with maximal dilatation of the tubular mid-ascending aorta, particularly at the greater curvature (convexity), with concomitant mild dilatation of the aortic root and proximal arch. Fazel et al. have suggested a classification for bicuspid aortopathy based on four distinct patterns of aortic dilation in patients with BAV (2) (Table 1).

**Table 1:** Fazel classification of aortic dilation in patients with BAV based on distinct patterns of aortopathy (2).

**Cluster**	**Aortic dilation pattern**
I	Aortic root
II	Tubular portion of the ascending aorta
III	Tubular portion of ascending aorta and transverse aortic arch
IV	Diffuse: root, ascending aorta, proximal aortic arch

## How Have the Clinical Guidelines Evolved Over the Years?

Prior to the most recent guidelines (3, 4) (Table 2), BAV patients were offered aortic resection primarily based on maximal aortic dimension and the rate of aortic expansion. These strategies were similar to those for patients with proven genetic aortopathies such as Marfan syndrome (5). Practices were also mostly based on surgeon and institutional experiences. However, recent clinical studies suggest that BAV patients do not behave like Marfan patients, providing a compelling case for more conservative treatment strategies. It was found that after aortic valve replacement without concomitant aortic resection, progression of aortopathy and aortic complications in long-term follow-up was attenuated in BAV patients as compared to those with Marfan syndrome, and more similar to tricuspid valve patients (6). At the present time, clinical data do not support surgical approaches for BAV aortopathy to match those with a documented connective tissue disorder. Therefore, further investigations into the role of hemodynamics to guide resection strategies are warranted. More recent clinical guidelines reflect these novel insights and are more conservative with respect to prophylactic resection as the primary indication for surgery (7). In the latest consensus guidelines targeted specifically for BAV patient, The American Association of Thoracic Surgery recommended repair of the aorta and aortic root when aortic diameter was >55 mm in patients without risk factors (3). European guidelines from 2017, which are provided for all valvular diseases, recommend surgery when aortic diameter is >50 mm in patients with BAV (4).

**Table 2:** The American Association for Thoracic Surgery consensus guidelines on bicuspid aortic valve–related aortopathy (3).

**Recommendation**	**Class/level of evidence**
Repair of the ascending aorta/root is recommended when the aortic diameter is ≥55 mm in patients without risk factors.	I/B
Repair of the ascending aorta/root is recommended when the aortic diameter is ≥55 mm in patients without risk factors.	IIa/B
Repair of the ascending aorta/root should be performed when the aortic diameter is ≥50 mm in patients with risk factors.	IIb/C
Concomitant repair of the ascending aorta/root should be performed when the aortic diameter is ≥45 mm in patients undergoing cardiac surgery	IIa/B
Repair of the aortic arch is recommended in patients with an aortic arch diameter of ≥55 mm.	I/B
Concomitant repair of the aortic arch should be performed in patients undergoing cardiac surgery with an aortic arch diameter of ≥50 mm.	IIa/C
Concomitant repair of the aortic arch may be performed in patients undergoing cardiac surgery with an aortic arch diameter of ≥45 mm, provided the patients are at low surgical risk and operated on by an experienced aortic team with established surgical results.	IIb/C
Concomitant repair of the aortic arch may be performed in patients undergoing cardiac surgery with an aortic arch diameter of ≥45 mm, provided the patients are at low surgical risk and operated on by an experienced aortic team with established surgical results.	I/B

Basic and translational research has informed the development of Guidelines. However, it is important to note that a limitation of these guidelines is that they have also been proposed based on studies reporting on diameters of already ruptured and/or dissected aortas. There is evidence that dissection itself leads to immediate aortic diameter increase of 30% for ascending and 25% for descending aorta (8, 9). Thus, patients who present with a type A aortic dissection and an ascending aorta diameter of 5.5 cm probably had a diameter of <4.0 cm prior to dissecting.

## What is the Underlying Cause of Bicuspid Aortopathy?

There is ongoing debate regarding the etiology of BAV aortopathy. It was previously believed that aortopathy observed in patients with a bicuspid aortic valve was similar to aortic pathology associated with a diseased tricuspid aortic valve (TAV). This was thought to be secondary to the turbulent blood out-flowing from a stenotic aortic valve. In the 1990 and 2000s, several observations led investigators to think that a strong genetic role contributed to bicuspid aortopathy and that the risk of acute aortic complications was substantially increased in this patient population (10). Although not universally seen in BAV patients, the NOTCH signaling pathway has been implicated in most studies as a genetic cause for BAV and bicuspid aortopathy (11, 12). As technology advances, our understanding of the genetic contributors to BAV and bicuspid aortopathy will improve.

There is a growing body of evidence supporting valve-related hemodynamics as an underlying cause of bicuspid aortopathy (13). Using four dimensional (4-D) flow MRI to map blood flow through the valve and aorta, normally-functioning bicuspid aortic valve patients were observed to have disturbed ascending aortic flow and increased regional hemodynamic stresses (14). Our group and others have also shown that aortic cusp fusion patterns lead to distinct orientations of eccentric flow jets (15–17), which in turn may result in differential distributions of aortic wall shear stress (15, 16), and subsequent focal flow-induced vascular remodeling (17). Propagation patterns of transvalvular flow are not uniform in BAV patients with the same cusp fusion morphology (15–18). Therefore, it is suggested that other parameters, such as the subvalvular apparatus and the geometric orientation of residual aortic valve orifice, could play an important role in directing vascular remodeling, and hence potentiating aortopathy (18). In reality, both genetic and hemodynamic theories probably coexist, and it is logical to speculate that valve-related hemodynamics may exacerbate disease progression in a genetically susceptible aorta. A better appreciation of regional hemodynamics using advanced imaging tools in individual patients could allow for more individualized resection strategies and improved outcomes for this heterogeneous disorder (14).

## Does the Etiology of Bicuspid Aortopathy Have Any Clinical Implications on Patient Care or Risk Assessment?

Since there is no consensus on the exact etiology of bicuspid aortopathy, there are highly variable practices in the surgical management of BAV patients (10). Those who believe genetics to be the primary contributor to aortopathy have advocated more aggressive approaches with respect to the timing and extent of aortic resection: early and wide surgical resection. While those who attribute lesser significance to genetics, and acknowledge aberrant fluid dynamics to play a sizeable role in the development of aortopathy, promote a more conservative management approach for these patients.

## Does the Pattern of Aortic Valve Cusp Fusion Help Predict the Risk of Aortic Complications?

Bicuspid aortic valve (BAV) is the most common congenital cardiac pathology, affecting 1–2% of the general population (19). Different classifications have been proposed for BAV; however the most common categorization is described by Sievers (20). Sievers type 0 BAV has no raphe and 2 valve cusps; type 1 BAV has a single raphe and 2 valve cusps; type 2 BAV has 2 raphes and 2 valve cusps. Bicuspid aortic valve morphology with fusion of the right-left coronary cusps (i.e., Sievers type I, R/L) and right-non-coronary cusp (Sievers type I, R/N) represent the two most common BAV morphologies, accounting for ~75 and 20% of clinical presentations, respectively (20). A greater prevalence of female patients are associated with R/N fusion pattern (21–23).

Predicting the progressive course of aortopathy and its associated complications in BAV patients is challenging. An emerging predictor of risk is the pattern of valve cusp fusion. The cusp fusion location is variable in BAV patients and may influence the expression of the clinical aortopathy. The majority of patients, as in our case example, have fusion of the right and left aortic cusps. These patients typically present with dilatation of the tubular ascending aorta, particularly along its convexity, accompanied by varying degrees of aortic root dilatation. Patients with fusion involving the non-coronary cusp are more likely to have dilation of the ascending aorta, rather than the sinuses, which often extends higher into the transverse arch (15). However, literature has not been able to show a consistent association between BAV-morphology and the observed pattern of aortopathy (24–28). Classification schemes have been suggested for the expression of the aortopathy, there is no identifiable scheme that is widely adopted or well validated for risk prediction in current clinical practice (29). Efforts to define specific biomarkers to predict aortic risk have also been investigated, but none have been widely adopted for clinical use as of yet (30).

## How is Bicuspid Aortopathy Associated With Adverse Aortic Complications?

Bicuspid aortopathy is associated with a constellation of poor clinical outcomes. When compared to a normal trileaflet aortic valve, complications of the bicuspid aortic valve itself are more common; including valvular stenosis, regurgitation, or infection. Dilatation of any or all segments of the proximal aorta is present in ~50% of individuals with a congenital bicuspid aortic valve, and severe aneurysmal aortic dilation may develop in some patients (18). It is believed that ascending aortic aneurysms occur at a frequency of 1 in 100 BAV patients per year (29). Bicuspid aortopathy is usually progressive and its presence increases the risk of unfavorable clinical events such as aortic rupture and dissection: 1 of every 1,000 BAV patients will experience aortic dissection per year (31, 32). This infers a low overall incidence of aortic rupture/dissection for patients with BAV, but the actual risk is increased as compared to patients with tricuspid aortic valves. Despite the low incidence of dissection, the associated mortality and morbidity is high when it occurs.

**Figure 2:**
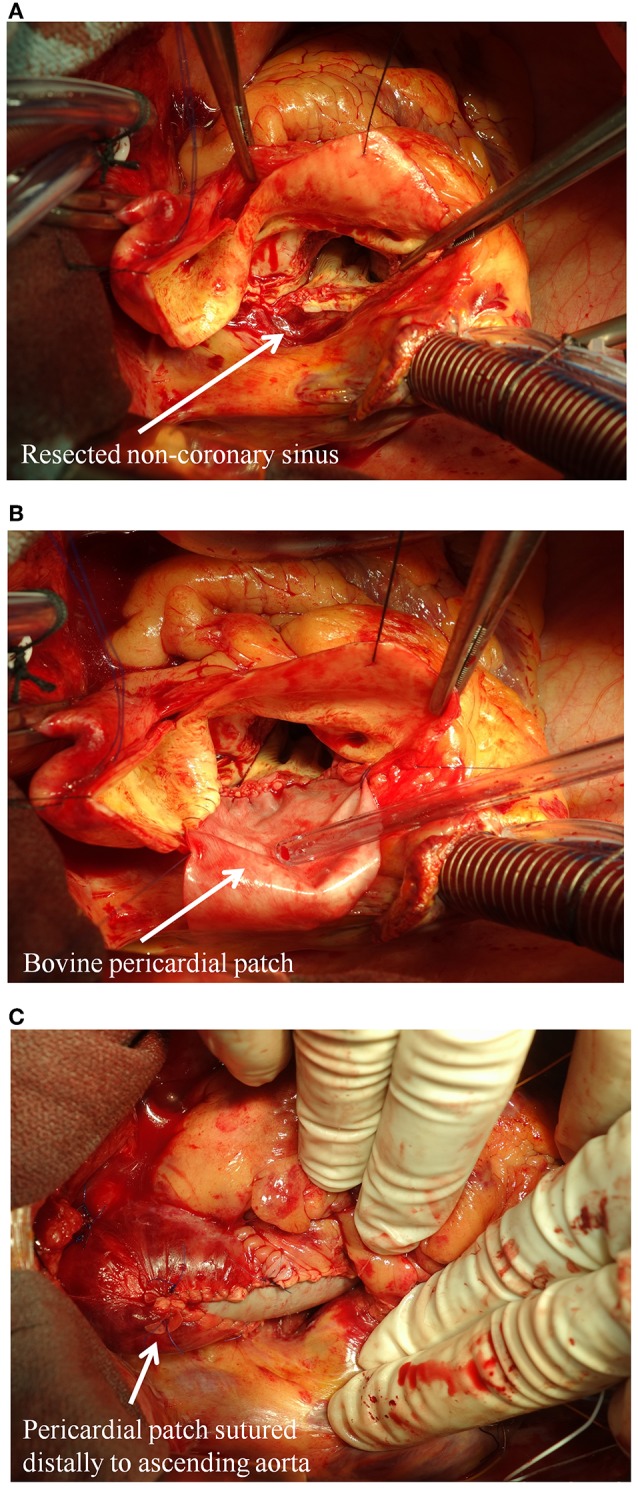
Partial aortic root repair with selective replacement of the non-coronary sinus in BAV patients. **(A)** The dilated non-coronary sinus is resected; **(B)** the non-coronary sinus is replaced with a bovine glutaraldehyde-fixed pericardial patch; **(C)** view of the sewn in patch.

Although found less commonly, patients with aortic regurgitation and isolated root dilatation may be at a higher risk of aortic events compared to other patterns of aortopathy (33). Root dilation is often asymmetric and more often affects the non-coronary sinus of Valsalva. Maximal aortic diameter >45 mm and rapid progression (>5 mm of diameter expansion per year) is associated with an increased risk of rupture/dissection. Surgery is most often necessary for valve failure, but prophylactic replacement of the ascending aorta is performed in ~25% of patients (within a 25 years span from time of diagnosis) based on contemporary resection guidelines (32). This strategy has yielded excellent outcomes, highlighting the significance of close surveillance of the aortopathy combined with timely surgical referral when indicated (34). Although more clinical studies are warranted, some groups have also suggested that partial aortic root repair, with selective replacement of the non-coronary sinus, can be a useful technique for BAV patients, as it avoids the risk of coronary manipulation (Figure 2) (35).

## What is the Optimal Modality and Timing of Imaging in Assessing Aortic Risk?

Similar to patients with trileaflet aortic valve disease, those with BAV require transthoracic echocardiography to assess aortic valve morphology and function. Using this modality the architecture of the aortic sinuses and proximal ascending aorta should also be carefully assessed. When the maximal diameter is <45 mm or the shape of the ascending aorta or root appears abnormal, more advanced imaging with CT or MR angiography is useful. In most cases it is difficult to assess the extent of aortopathy using echocardiography, particularly in the distal segments of the aorta (36). An echocardiogram is also not optimal for evaluating growth patterns and root anatomy, especially the non-coronary sinus. If echocardiography is used to assess the aortic root and ascending aorta, as per the 2017 European guidelines, measurements should be made at four levels: annulus, sinus of Valsalva, sinotubular junction, and tubular ascending aorta. Measurements are taken in the parasternal long-axis view from leading edge to leading edge at the end of diastole, except for the aortic annulus, which is measured in mid systole (4).

However, CT and MRI have better spatial resolution than echocardiography and the images can be reconstructed in 3 dimensions, providing a better appreciation of the aortopathy, aortic root, and growth patterns. CT remains the gold-standard imaging modality, but the long-term risks of cumulative radiation exposure over years of surveillance must be considered. If CT angiography is used to assess the aorta, it is important to note that the true aortic diameter is one which includes the aortic wall. The 2017 European guidelines have also recommended diameter size adjustment for calculating aortic dimensions when using different imaging modalities (4). The calculation of indexed values has been recommended to account for body size (37).

In serial imaging of the bicuspid aorta, it is crucial to document the rate of aortic expansion, the location, and the size of maximal aortic diameter. These parameters will guide the decision to proceed with prophylactic aortic resection. As such, patients with increased risk factors and/or substantial baseline aortopathy (maximal diameter > 45 mm) should have annual assessments. Aortic growth rate can be highly variable between patients, but typical rates of growth are in the 0.1–0.5 mm per year range. Growth rates > 3 mm per year are considered high risk and surgery should be strongly considered. Patients with mild degrees of aortopathy and low rates of progression could be assessed on a less frequent basis, such as every 2 years. Surveillance and serial imaging may not be indicated for patients who are not surgical candidates due to either increased surgical risk or patient preference. There are no clear data to guide the optimal timing of aortic surveillance in patients with BAV, so clinicians must assess each case individually. The recommended imaging follow-up plan should reflect the risk profile and likelihood of surgery for each individual patient. An appreciation of key risk factors, which include genetics and peri-operative comorbidities, is important for clinicians to consider for personalized patient care decisions (38, 39). A team approach with specialized cardiologists, surgeons, and imaging experts can be useful for patients with bicuspid aortopathy.

## When Should this Patient Have his Aorta Prophylactically Resected?

Before recommending and pursuing an invasive management plan, it is imperative to fully assess the patient. Subjecting an asymptomatic patient to invasive prophylactic aortic resection requires a careful deliberation of the risks of surgery weighed against the risks of a long-term aortic complication. In the past (2008 guidelines), surgical resection of the aorta was indicated when the maximal aortic diameter was 50 mm or greater (6). The surgical size threshold has become more conservative reflecting more recent data that suggest the risk of aortic dissection is low in patients with bicuspid aortopathy (24, 40, 41). These studies corroborate the findings of a retrospective comparison of over 13,000 patients, where it was clearly shown that the risk of aortic complications in long-term follow for BAV patients was much closer to control patients than those with Marfan syndrome (7). At the present time, surgery is recommended for bicuspid aortopathy when the maximal aortic diameter is 55 mm or greater in patients who do not have any high risk characteristics (36). This size threshold is recommended for all levels of the aorta, including the aortic root segment, and should not be adjusted based on body size indices. For example, root phenotype, valvular pathology, and patient age should be considered (38). The lower threshold of 50 mm would be appropriate in those with risk factors for aortic complication, such as rapid growth, concomitant aortic valve disease, concurrent connective tissue, or genetic syndromes. This, however, may not be a prudent approach in those at increased surgical risk. The size range of 50–55 mm is considered the “gray zone,” and “centers of excellence” may adopt a more aggressive approach within that range, compared to sites with smaller surgical volumes. The above recommendations are based on the premise that aortic complications, representing a long term risk that will increase with time, will be avoided by elective aortic replacement performed at a low mortality rate (≤ 1% at experienced centers). It is reassuring that in experienced surgical programs the risk of surgery is low and long-term outcomes are excellent when clinical guidelines are closely followed (42). As reflected in differences in European vs. North American guidelines, the evidence supporting size thresholds are limited and decisions must be made on an individualized basis (4). The final decision to proceed with a prophylactic aortic resection in patients with BAV aortopathy should involve an informed discussion between the cardiologist, surgeon, and patient.

The primary indication for surgery in patients with BAV also influences the size threshold. The majority of patients with BAV will have surgery to repair or replace the bicuspid valve. In patients who are candidates for surgery based on valve dysfunction or infection, it is recommended to replace the ascending aorta when >45 mm in maximal diameter. While size thresholds should be considered at all levels of the aorta, it is important to appreciate that the aortic sinus segments are typically 5 mm larger than the ascending aorta, and often asymmetrically dilated in many patients with BAV. This results in resection being more complicated when the coronary arteries are reimplanted. Although more robust clinical outcomes data is needed, as suggested above, partial root repair with selective replacement of the non-coronary sinus can be a useful technique for these patients (35). Intraoperative judgment is critical in these clinical scenarios and consideration should be given to more conservative approaches in selected cases.

In the case example, with an ascending aorta of 52 mm and no clear indication for primary valve surgery with only moderate aortic valve dysfunction, prophylactic aortic resection would not be indicated in the absence of additional risk factors. As highlighted, however, the rate of growth is an important risk factor. Furthermore, in many cases serial imaging may not be available to determine the rate of growth. In this situation it is reasonable to repeat imaging at an early interval, such as 6 months. In the case example, aortic expansion is rapid at 5 mm per year, thus prophylactic aortic resection should be strongly considered.

## How Should this Patient's Aorta be Resected? How Much Should be Resected? How Will the Surgeon Make This Decision?

The extent of prophylactic surgical resection is often surgeon-dependent and evidence for the extent of resection is unclear. A survey of surgical approaches to bicuspid aortopathy showed a high variability that may be related to a surgeon's assessment of long-term risk and cause of the aortopathy (5). Resection of the aortic root and proximal aortic arch requires much more complex and extensive surgical intervention when the coronary arteries and branches of the aortic arch are involved. Although there is a paucity of robust clinical data, studies have not supported routine resection of the proximal aortic arch in patients with bicuspid aortopathy (43–45). Nevertheless, at high volume and expert centers, a more aggressive approach may be undertaken in intervening upon the aortic root and hemiarch. Some studies have investigated the outcome of an untreated aorta when only the bicuspid aortic valve is replaced (32, 46–53). None have shown a significantly increased risk in patients who have only had their aortic valve is replaced. Similarly, literature has not been able to consistently demonstrate that an un-replaced root dilates if left intact at the time of aortic valve replacement for patients with bicuspid aortic valves (54).

Approaches for the extent of the resection should reflect recent data indicating that long-term risk is low. For individuals with dilatation of tubular ascending aorta, surgical options include: supracoronary replacement of the ascending aorta; or, if significant aortic valve dysfunction and aortic root dilatation are also present, aortic valve, root, and ascending aortic replacement is indicated. In patients with dilated ascending aorta and aortic arch, surgical options may include aortic valve replacement with supracoronary replacement of the ascending aorta and hemi-arch replacement that may involve deep hypothermic circulatory arrest. In individuals with isolated root involvement, surgical options may include aortic valve and root replacement using a composite valved conduit (i.e., a Bentall procedure), or a partial root repair. In expert hands, a valve-sparing operation may be carried out, where the proximal ascending aorta and root segment can be replaced leaving the bicuspid valve intact (10). In most cases, surgical repair for bicuspid aortopathy can be achieved with excellent results in experienced centers.

## Case Summary

The patient did not have a primary indication for valve surgery and as such, did not need aortic intervention based on size alone (where guidelines recommend aortic size >5.5 cm). However, after careful consultation and communication with both his cardiologist and surgeon, the rapid aortic growth rate (>5 mm per year in this patient's case) combined with a low operative risk for aortic resection prompted replacement of the ascending aorta using a supracoronary graft. The aortic root and sinus segments were only mildly dilated (<4.5 cm), as was the hemi-arch, so these aortic regions were spared from resection. The aortic valve was not the primary indication for surgery but was replaced at the time of aortic resection given the findings of moderate aortic regurgitation in association with evidence of progressive left ventricular dilatation and mild exercise limitation.

## Author Contributions

AFH was the primary author, responsible for the manuscript's inception and preparation along with PF. CF provided insight into the surgical management of bicuspid aortopathy. SV provided expertise for the management of BAV and bicuspid aortopathy.

### Conflict of Interest Statement

The authors declare that the research was conducted in the absence of any commercial or financial relationships that could be construed as a potential conflict of interest.

## Footnotes

^1^The case presentation is entirely fictional.
